# EGFR806-CAR T cells selectively target a tumor-restricted EGFR epitope in glioblastoma

**DOI:** 10.18632/oncotarget.27389

**Published:** 2019-12-17

**Authors:** Ali C. Ravanpay, Juliane Gust, Adam J. Johnson, Lisa S. Rolczynski, Michelle Cecchini, Cindy A. Chang, Virginia J. Hoglund, Rithun Mukherjee, Nicholas A. Vitanza, Rimas J. Orentas, Michael C. Jensen

**Affiliations:** ^1^ Ben Towne Center for Childhood Cancer Research, Seattle Children’s Research Institute, Seattle, WA, U.S.A; ^2^ University of Washington, Department of Neurological Surgery, Seattle, WA, U.S.A; ^3^ University of Washington, Department of Neurology, Seattle, WA, U.S.A; ^4^ Center for Integrative Brain Research, Seattle Children’s Research Institute, Seattle, WA, U.S.A; ^5^ Center for Clinical and Translational Research, Seattle Children’s Research Institute, Seattle, WA, U.S.A; ^6^ University of Washington, Department of Pediatrics, Seattle, WA, U.S.A; ^7^ University of Washington, Department of Bioengineering, Seattle, WA, U.S.A

**Keywords:** glioblastoma, CAR T cell, EGFR, immunotherapy, mAb806

## Abstract

Targeting solid tumor antigens with chimeric antigen receptor (CAR) T cell therapy requires tumor specificity and tolerance toward variability in antigen expression levels. Given the relative paucity of unique cell surface proteins on tumor cells for CAR targeting, we have focused on identifying tumor-specific epitopes that arise as a consequence of target protein posttranslational modification. We designed a CAR using a mAb806-based binder, which recognizes tumor-specific untethered EGFR. The mAb806 epitope is also exposed in the EGFRvIII variant transcript. By varying spacer domain elements of the CAR, we structurally tuned the CAR to recognize low densities of EGFR representative of non-gene amplified expression levels in solid tumors. The appropriately tuned short-spacer 2nd generation EGFR806-CAR T cells showed efficient in vitro cytokine secretion and glioma cell lysis, which was competitively blocked by a short peptide encompassing the mAb806 binding site. Unlike the nonselective Erbitux-based CAR, EGFR806-CAR T cells did not target primary human fetal brain astrocytes expressing wild-type EGFR, but showed a similar level of activity compared to Erbitux-CAR when the tumor-specific EGFRvIII transcript variant was overexpressed in astrocytes. EGFR806-CAR T cells successfully treated orthotopic U87 glioma implants in NSG mice, with 50% of animals surviving to 90 days. With additional IL-2 support, all tumors were eradicate without recurrence after 90 days. In a novel human induced pluripotent stem cell (iPSC)-derived teratoma xenograft model, EGFR806-CAR T cells infiltrated but were not activated in EGFR+ epidermal cell nests as assessed by Granzyme B expression. These results indicate that EGFR806-CAR T cells effectively and selectively target EGFR-expressing tumor cells.

## INTRODUCTION

Solid tumor treatment with CAR T cells is currently limited by the paucity of cell surface targets that are tumor restricted, broadly expressed throughout the tumor, and integrally associated with tumorigenicity. Designing CARs to minimize on-target off-tumor toxicity is an important consideration in CAR design given the potentially serious consequences of targeting normal tissue [1]. Central nervous system (CNS) tumors continue to pose an exceptional challenge in oncology due to the difficulty of delivering drugs through the blood-brain barrier, and the high morbidity of radiation and surgical approaches. The need for better therapies is particularly urgent for malignant gliomas, as well as other brain tumors that are refractory or recur after standard therapy. Currently, 5-year survival rates range from less than 5% for adult glioblastoma [2, 3] to 25% or less for recurrent or refractory medulloblastoma [4]. EGFR amplification, overexpression, or mutation is present in approximately half of glioblastomas and other malignant CNS tumors, including ependymoma and medulloblastoma, in both children and adults [5–12]. EGFR-targeted interventions such as tyrosine kinase inhibitors have had limited success in CNS tumors, likely due to difficulty accessing the tumors and tumor evasion of these pathway-based drugs [13, 14]. CAR T cells targeting EGFRvIII, a truncation mutant of EGFR that is common in glioblastoma, were safe but unable to eradicate the tumor, likely due to nonuniform expression of the target protein and antigen escape [15, 16].

To construct a CAR targeting EGFR-expressing CNS tumors, we chose the mAb806 scFv moiety due to its unique binding characteristics. mAb806 was initially raised against the EGFRvIII variant, but also binds to full-length EGFR that is expressed as a result of gene amplification [17, 18]. However, it does not recognize EGFR on normal cells [19]. This tumor specificity is likely due to changes in steric accessibility of the mAb806 epitope between tumor-expressed and wild type EGFR [20]. The mAb806 epitope is located on extracellular domain II of EGFR [21], and is inaccessible to the antibody during normal conformational states of the receptor, both the tethered, monomeric, unbound form and the untethered, dimerized, ligand bound form [22]. However, posttranslational modifications during overexpression of EGFR sterically expose the mAb806 binding site [21, 23]. In addition, multiple EGFR mutations found in glioblastoma, including EGFRvIII, produce a conformational change in the extracellular domain that may promote pro-growth signaling and also renders the mAb806 epitope accessible [20].

The mAb806 antibody has been tested in clinical trials for EGFR-overexpressing tumors, and showed specific binding to tumor tissue, including malignant glioma, with only mild gastrointestinal and dermatologic side effects [24]. A humanized version of the antibody is now in clinical trials as an antibody-drug-conjugate targeting recurrent glioblastoma [25]. We chose intracranial delivery of the CAR T cells as this approach effectively places the cells near their target, lowers the risk of side effects outside the CNS, and has shown promise in clinical trials for glioblastoma [26].

EGFR signaling plays an important role during brain development [27, 28], and has been shown in animal models to be involved in CNS injury, repair, and adult neurogenesis [29, 30]. Although no EGFR expression has been conclusively demonstrated in postnatal human brain [31–33], there remains theoretical concern that expression could be reactivated in the setting of cancer treatment, or that intracranially injected CAR T cells could emerge into the systemic circulation. Therefore, we tested tumor-specificity on physiologically EGFR-expressing fetal human astrocytes. In addition, we developed an *in vivo* teratoma assay to measure CAR T cell infiltration and activation in implanted human iPSCs that were allowed to differentiate into multiple tissues tissue types, including EGFR-expressing epithelia [34]. We show that second-generation EGFR806-CAR T cells with a short spacer can eradicate malignant glioma in a xenograft mouse model via intracranial delivery, and that CAR T cell activation is specific to tumor-expressed EGFR.

## RESULTS

### EGFR806-CAR with extracellular short spacer shows efficient EGFR+ tumor lysis and cytokine production

**Figure 1:**
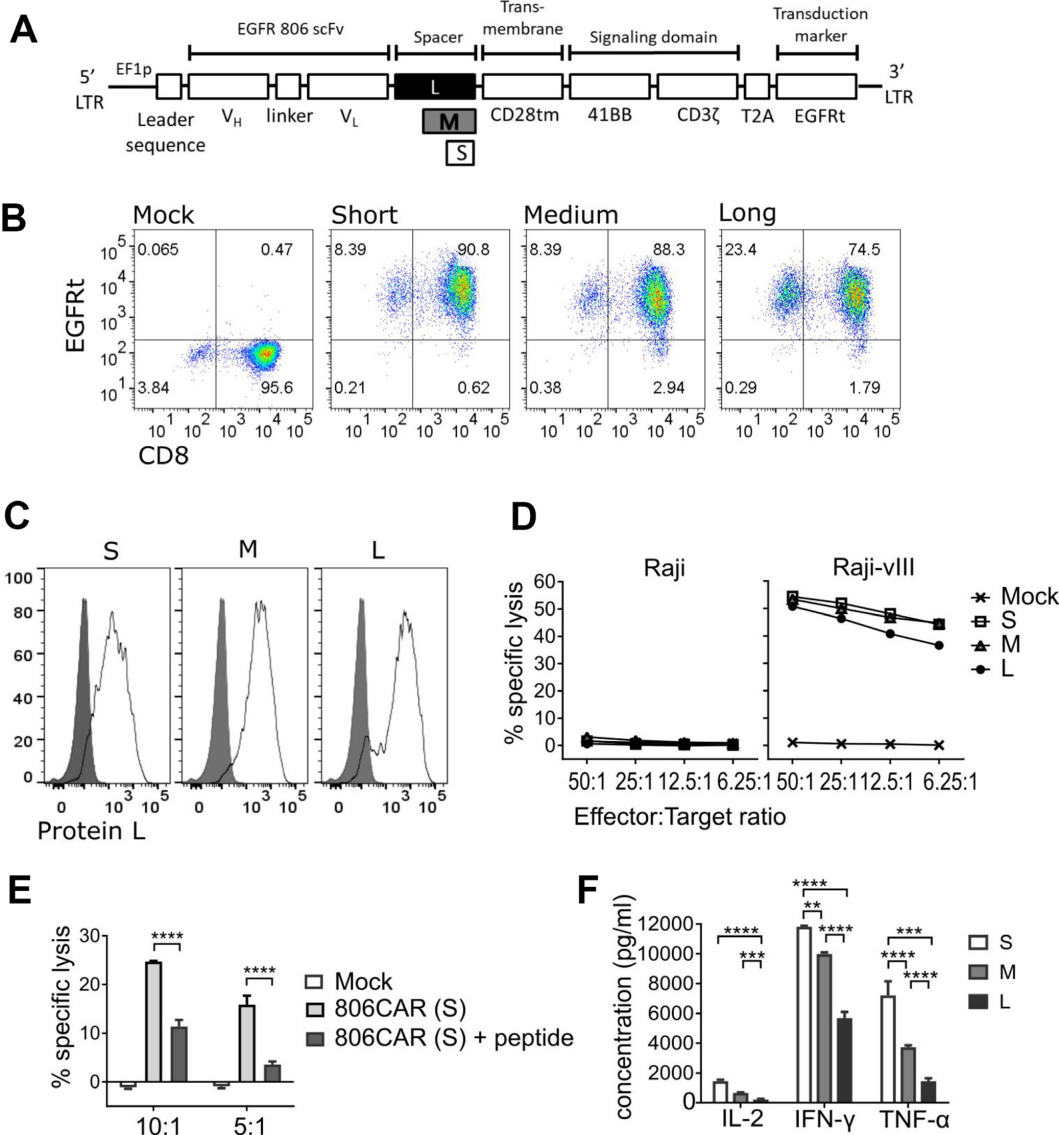
EGFR806-CAR T cells effectively target EGFR-expressing glioma cells *in vitro*. (**A**) Schematic of 2^nd^ generation EGFR806-CAR extracellular domain variants. Spacer variants: Short (S) – IgG4-hinge; Medium (M) – IgG4-hinge-CH3; Long (L) – IgG4-hinge-CH2-CH3. (**B**) EGFRt expression by flow cytometry showing CAR expression in CD8^+^ T_CM_ transduced with short, medium or long spacer 2nd generation EGFR806-CAR lentivirus and selected for EGFRt expression. Mock transduced cells were unselected. Representative data for one donor are shown, and all donors (*N* = 3) yielded >90% EGFRt positive T cells after the selection step. (**C**) Protein-L was used to label the scFv portion of the CAR, demonstrating surface expression. Representative data from one donor. (**D**) EGFR806-CAR T cells kill EGFRvIII-expressing Raji (Raji-vIII) cells but not untransduced Raji cells in a 4-hour chromium release assay. The x-axis shows the ratio of effector: target cells. (**E**) An EGFR aa. 287-302 peptide, which encompasses the mAb binding epitope, inhibits short-spacer EGFR806-CAR T cell lysis of Raji-vIII cells. The x-axis shows the ratio of effector: target cells. (**F**) Cytokine levels in supernatants obtained from 24hr co-cultures of EGFR806-CAR T cells expressing extracellular spacer variants and Raji-vIII cells at a 2:1 ratio. Cytokine data from three independent experiments was analyzed by Student *t* test. Error bars represent SEM. All panels: S, short spacer; M, medium spacer; L, long spacer. ^**^ *P <* 0.01; ^***^ *P <* 0.01; ^****^ *P*.001.

To target EGFR-positive tumors, we engineered a 2nd generation CAR construct consisting of an extracellular binding domain derived from mAb806, a 4-1BB-z intracellular signaling domain, and truncated EGFR (EGFRt) to serve as a transduction marker and ablation target (Figure 1A). The EGFRt fragment does not contain the mAb806 binding site and thus is not recognized by the EGFR806-CAR [21]. Since the length of CAR extracellular spacer domains has been shown to affect CAR mediated cellular cytotoxicity [35], we addressed the functional impact of spacer lengths on EGFR806-CAR T cell activity by engineering CARs with modular spacer domains designated short (S, IgG4hinge alone), medium (M, IgG4hinge-CH3), and long (L, IgG4hinge-CH2-CH3) (Figure 1A). Thus, we purified CD8^+^CD45RO^+^CD62L^+^ central memory T cells (CD8^+^ T_CM_) [36] and transduced them with lentiviral vectors containing S-, M-, or L-spacer EGFR806-CARs. Initial transduction efficiencies based on EGFRt expression ranged from 74–90%, and transgene positive T cells were enriched to uniform purity (> 95%) by EGFRt selection (Figure 1B). Similar levels of surface and total CAR expression were confirmed by Protein-L staining (87–95%; Figure 1C) and a-CD3z western blot analysis (data not shown) respectively.

The *in vitro* lytic activity of the EGFR806-CAR T cells, as determined by chromium release assay, was analyzed against Raji cells transduced with an EGFRvIII construct (Figure 1D). Each EGFR806-CAR version conferred similar levels of specific lysis against EGFRvIII-expressing Raji cells, but did not recognize parental Raji (EGFR-negative) targets. EGFR806-CAR specificity was further verified by an inhibition study using an EGFR-derived peptide that contains the putative epitope of mAb806 (Figure 1E). At a concentration of 55 mM (100 mg/ml) [37], the soluble peptide inhibited the lytic capacity of short spacer EGFR806-CAR T cells by 54-78%, depending on effector to target (E: T) ratios. Although the tumor lytic capacity was similar for the three spacer variants, the short spacer CAR induced the most robust effector cytokine production upon tumor recognition, with 4.6- (*P <* 0.0001), 3.8- (*P <* 0.0001), and 3.1-fold (*P*=0.0001) higher IL-2, IFNg, and TNFa levels, respectively, compared to long spacer; and 2.2- (*P* > 0.05), 1.2- (*P <* 0.01) and 1.9-fold (*P <* 0.0001) higher IL-2, IFNg, and TNFa levels compared to medium spacer CAR (Figure 1F). All three cytokines were undetectable when CAR T cells were cultured in the absence of target, or with parental Raji cells (data not shown).

### EGFR806-CAR T cells effectively lyse low-EGFR expressing glioblastoma cell lines independent of EGFRvIII expression

**Figure 2:**
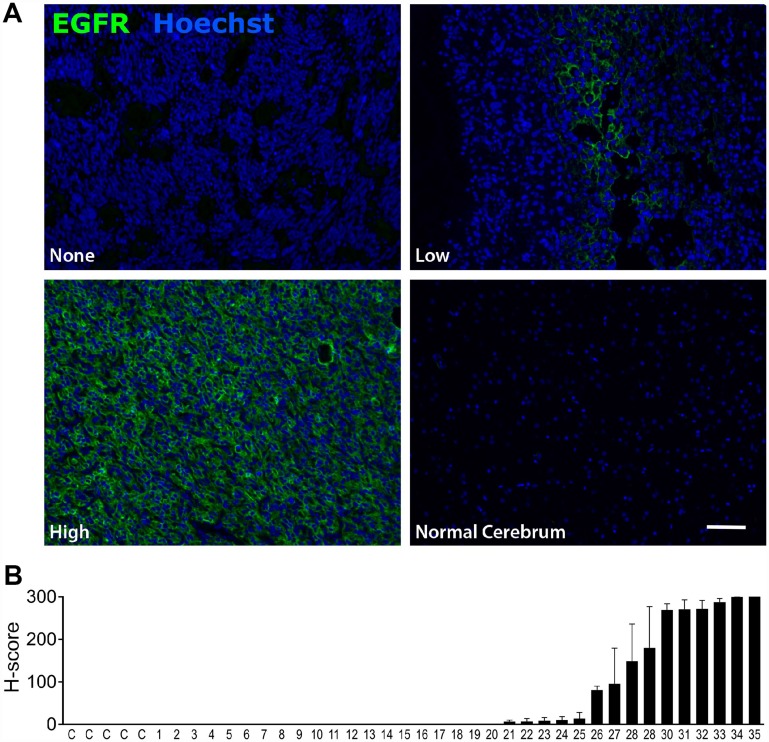
EGFR is expressed in glioblastoma but not in normal brain. (**A**) Representative immunofluorescent labeling for EGFR expression in human glioblastoma samples (top row and bottom left panels) and normal brain (bottom right panel). Scale bar 20μm. Images were acquired at 20 ×. (**B**) EGFR expression in normal brain (C=control) and 35 malignant glioma samples (1–35). Bars show mean EGFR immunofluorescence as quantified by H score. Error bars show SEM for 2 replicates from individual patients.

**Figure 3:**
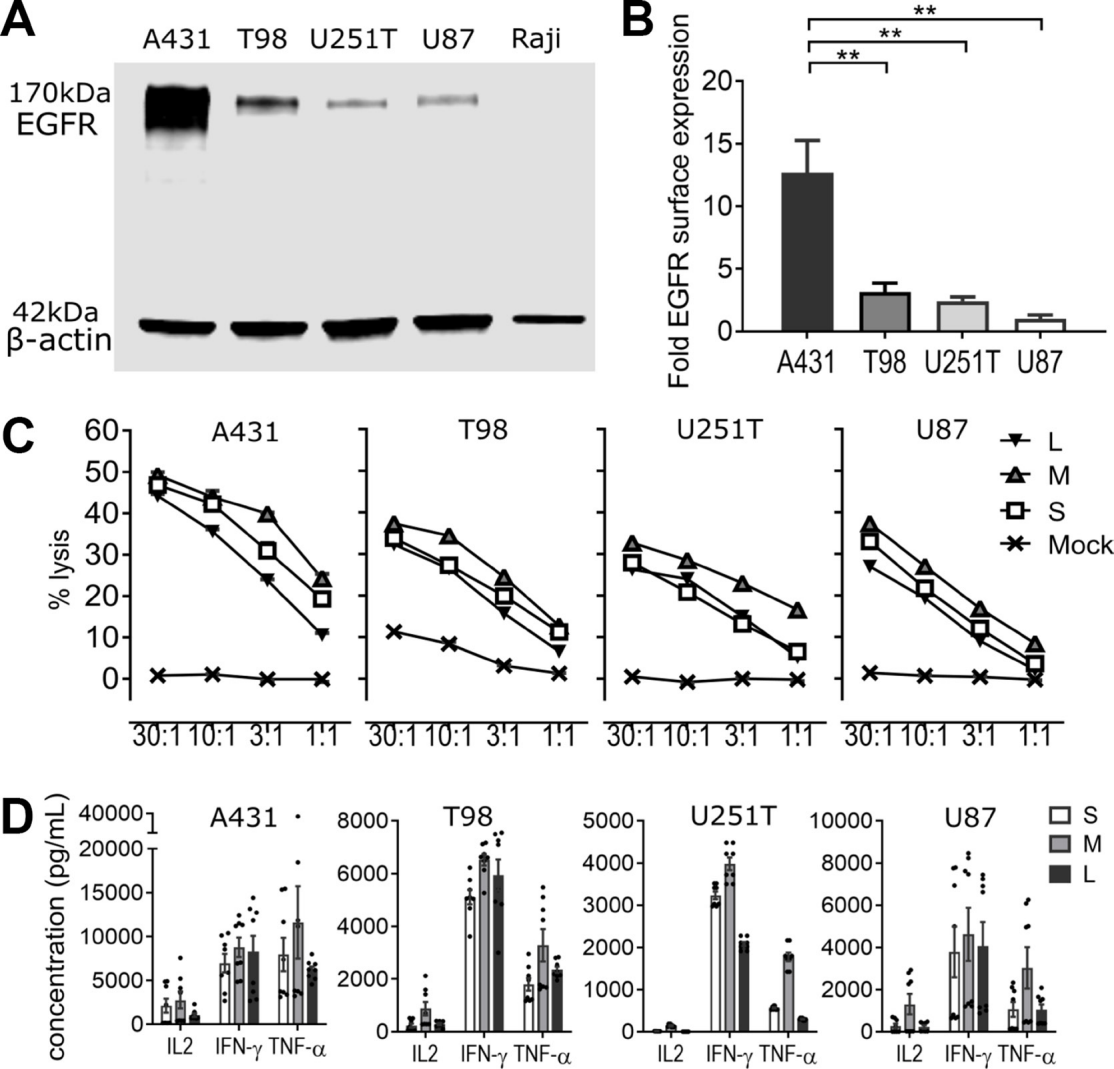
EGFR806-CAR T cells are selectively activated by tumor-specific EGFR expression *in vitro*. (**A**) EGFR quantification in tumor cell lysates via Western blot. Top: EGFR expression in tumor cell lines. Bottom: actin loading control. A431: EGFR^+^ squamous carcinoma cells (positive control); T98, U251T and U87: glioblastoma cell lines; Raji: EGFR^-^ Burkitt’s lymphoma cells (negative control). (**B**) Quantification of EGFR surface antigen expression by flow cytometry. The y-axis shows normalized EGFR expression relative to the lowest-expressing U87 cells, ± SEM of three independent experiments. (**C**) Cytolytic capacity of EGFR806-CAR T cells expressing extracellular spacer variants against EGFR^+^ tumor cell targets in 4 hr chromium release assay. The x-axis shows the effector: target cell ratio. (**D**) Cytokine quantification of supernatants obtained from 24 hr co-cultures of EGFR806-CAR T cells and EGFR^+^ tumor targets at a 2:1 effector: target ratio. Each dot represents an individual replicate, bars show mean ± SEM. Data pooled from two separate experiments with CAR T cells from two different donors. S, short spacer; M, medium spacer; L, long spacer; Mock, untransduced CAR T cell control. ^**^ *P <* 0.01.

To validate EGFR as a suitable target for CAR based immunotherapy, we confirmed expression of EGFR on 35 separate malignant glioma samples via tumor tissue microarray. EGFR expression was high in 23% (H score >100), low (H score 5–100) in 20%, and absent in 57% of samples, and also absent in normal brain (Figure 2). We then used western blot and cell surface quantification of EGFR in three established glioblastoma cell lines (T98, U251T, and U87) to assess their ability to recapitulate tumor EGFR expression (Figure 3A, 3B). Epithelial carcinoma (A431) and Burkitt’s lymphoma (Raji) cell lines were used as positive and negative controls, respectively. U87 cells had the lowest level of EGFR surface expression, at 8% of the high-expressing A431 line (Figure 3B). EGFR was expressed on >99% of cells for all tumor cell lines (data not shown). We used RNA-Seq analysis to confirm the absence of EGFRvIII variant expression in the EGFR-positive cell lines, confirming that the activity of the EGFR806-CAR T cells in these cell lines is not mediated by binding to EGFRvIII (Table 1, Supplementary Figures 1 and 2). We then examined the *in vitro* lytic capacity of EGFR806-CAR T cells in the three human glioblastoma cell lines and A431 cells (Figure 3C). Similar to the Raji-vIII studies, we observed that the second generation EGFR806-CAR spacer variants mediated comparable levels of specific lysis at each effector: target ratio tested. Notably, cell targets with low surface EGFR expression relative to A431 were also efficiently lysed, although production of IL-2, TNFa and IFNg was lower when glioma cell lines were used as targets compared to A431 (Figure 3D). Based on these results, we selected the short spacer EGFR806 construct for further in-depth analysis. The short spacer CAR showed superior cytokine production against Raji-EGFRvIII cells, comparable activity against glioma cell lines, and minimizes lentiviral payload to allow the most efficient CAR transduction.

**Table 1:** EGFR and EGFRvIII expression in cell lines by RNASeq

Cell line	Unique loci	Multiple loci	Total reads	Mapping (%)	EGFR	EGFRvIII	EGFR %
A431	32,728,176	3,077,612	35,805,788	95.32	92856	4	0.259
T98	32,227,277	3,535,139	35,762,416	96.79	8465	5	0.024
U251T	25,629,699	2,680,718	28,310,417	92.20	2639	4	0.009
U87	28,414,376	2,596,026	31,010,402	92.44	6088	6	0.020
Raji-vIII	24,459,634	3,252,848	27,712,482	94.97	11	2783	0.010

Number of reads that align to unique as well as multiple genomic loci is given for 4 tumor cell lines and Raji cells transduced with EGFRvIII. The number of reads that map to endogenous EGFR and EGFRvIII is shown. The last column shows the fraction of total reads that map to EGFR (for A431, T98, U251T, and U87 cell lines) or EGFRvIII (for Raji-vIII control).

### Lower on-target off-tumor activity of EGFR806-CAR relative to Erbitux-CAR

**Figure 4:**
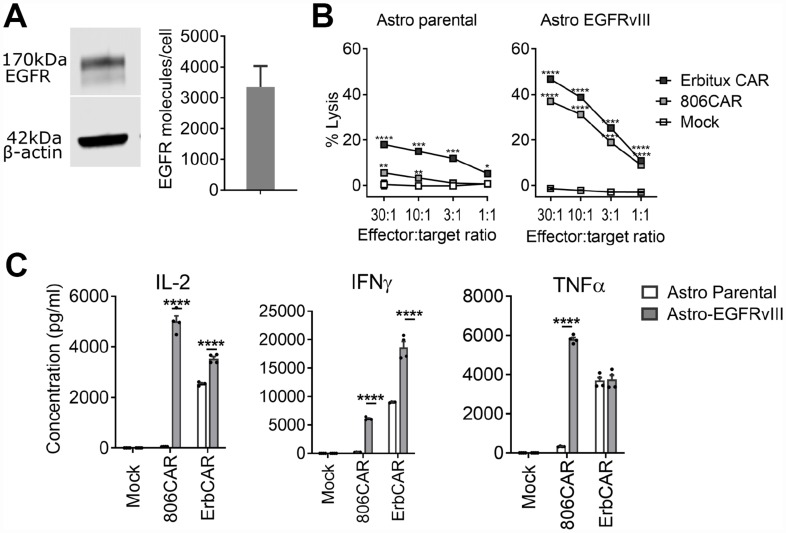
EGFR806-CAR T cells have lower activity against physiologically expressed EGFR compared to Erbitux-CAR. (**A**) Fetal human astrocytes express wild-type EGFR by Western blot (left) and surface expression is demonstrated by flow cytometry (right). (**B**) Lysis of fetal human astrocytes by EGFR806-CAR and Erbitux-CAR T cells in 4-hour chromium release assay. Left: untransduced fetal human astrocytes (Astro parental) are only minimally recognized by EGFR806-CAR T cells but are killed by Erbitux-CAR T cells. Right: after transduction with EGFRvIII (Astro EGFRvIII), fetal human astrocytes are killed efficiently by both effectors. Mock, untransduced CAR T cell control. Error bars show ± SEM for 3 replicate assays. (**C**) Cytokine production is stimulated by EGFRvIII-transduced, but not by wild-type EGFR expressing fetal human astrocytes when co-cultured with EGFR806-CAR T cells. Erbitux-CAR (ErbCAR) T cells show nonselective cytokine activation. Each dot represents an individual replicate, bars show the mean ± SEM of 4 replicates each. ^*^ *P <* 0.05; ^**^ *P <* 0.01; ^***^ *P <* 0.01; ^****^ *P* .001.

To test binding selectivity, we compared the EGFR806-CAR with an Erbitux-CAR, which binds EGFR expressed on both cancerous and normal tissues via a high-affinity binder derived from cetuximab [38]. Erbitux-CAR T cells have similar lysis capacity as EGFR806-CAR T cells against the glioma cell lines T98, U87, and U251T (data not shown). However, the 2^nd^ generation short spacer EGFR806-CAR T cells demonstrated selective cytotoxicity and cytokine release relative to Erbitux-CAR T cells when co-cultured with primary fetal human astrocytes, which express wild-type EGFR (Figure 4A). Untransduced fetal human astrocytes were killed by Erbitux-CAR T cells, while EGFR806-CAR T cells showed only minimal lysis even at high effector: target ratios (Figure 4B). However, when the primary human astrocytes were transduced to express EGFRvIII ectopically, they were lysed at similar rates by EGFR806-CAR T cells and Erbitux-CAR T cells (Figure 4B). Cytokine production showed a concordant pattern. There was negligible release of IL-2, IFNg, and TNFa when EGFR806CAR T cells were incubated with untransduced fetal human astrocytes (Figure 4C), while high levels of the same cytokines were released during lysis of EGFRvIII-overexpressing astrocytes. Erbitux-CAR T cells, in contrast, showed robust cytokine production with both untransduced and EGFRvIII-expressing astrocytes (Figure 4C), although both target cell lysis and cytokine production were higher with EGFRvIII astrocytes (Figure 4B, 4C). The difference in cytokine production between Erbitux-CAR and EGFR806-CAR may be due to differences in effective antigen density, where the Erbitux-CAR recognizes both overexpressed EGFRvIII and native EGFR, while the EGFR806-CAR is only activated by the overexpressed EGFRvIII. These results show that the lower on-target toxicity observed with primary fetal human astrocytes is not due to an inherent functional deficiency of the EGFR806-CAR. Instead, the EGFR806-CAR has higher specificity for pathologically expressed EGFR and EGFRvIII compared to native EGFR, which may translate to a more favorable *in vivo* toxicity profile compared to the Erbitux-CAR.

### Short spacer EGFR806-CAR T cells eradicate glioblastoma xenografts

**Figure 5:**
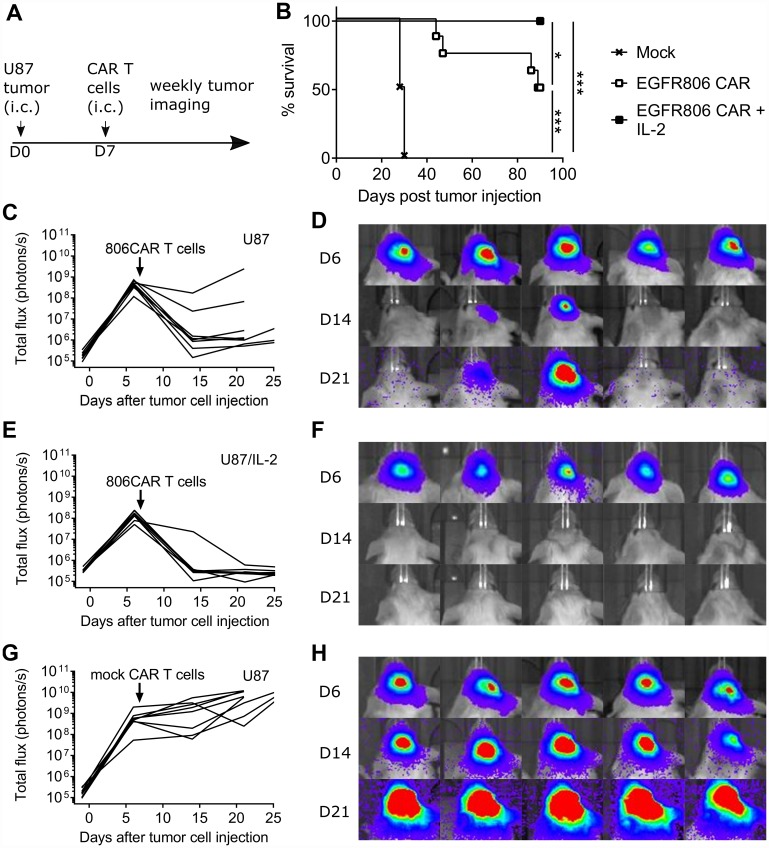
Short spacer second generation EGFR806-CAR T cells induce glioblastoma xenograft regression. (**A**) Experimental scheme. On day 0, NSG mice were intracranially (i. c.) injected with U87-ffluc cells with or without IL-2 overexpression. CAR T cells were delivered into the tumor bed on day 7, followed by weekly bioluminescence imaging. (**B**) Kaplan-Meier curve showing combined overall survival of mice treated with short spacer EGFR806 CAR T cells or mock transduced T cells. (**C**) Total tumor burden as expressed by total flux (photons/s) in individual mice bearing U87 intracranial tumors and treated with short spacer EGFR806-CAR T cells, with (**D**) showing representative bioluminescence images on days 6, 14, and 21 post treatment from the same group of animals. (**E**) Total tumor burden in individual mice bearing U87 intracranial tumors that overexpress IL-2, and treated with short-spacer EGFR806-CAR T cells, with (**F**) showing representative bioluminescence images from this group of animals. (**G**) Total tumor burden in individual mice bearing U87 intracranial tumors, treated with mock transduced T cells, with (**H**) showing representative images from this group. *N* = 8 for each group, pooled data from 2 experiments.

To confirm the *in vitro* anti-glioma activity of the EGFR806-CAR *in vivo*, we tested tumor killing capacity in a previously described orthotopic U87 glioma xenograft tumor model [39]. NSG mice were inoculated with the human U87 cell line by intracranial (i. c.) injection and seven days later treated with a single intratumoral dose of short spacer EGFR806-CAR T cells (Figure 5A, 5B). Control mice received mock transduced human donor T cells. EGFR806-CAR T cell treatment induced tumor regression in all animals (Figure 5C–5F). While all animals treated with control T cells had to be euthanized between day 28-30 (Figure 5G, 5H), 50% of EGFR806-CAR treated mice survived to the predetermined experimental end point of 90 days (*P*=0.0001, log rank test) (*N* = 8 for each group, pooled data from 2 separate experiments). This antitumor efficacy was markedly higher than in mice treated with T cells transduced with the medium or long spacer second-generation EGFR806-CAR or a third generation EGFR806-CAR with 4-1BB and CD28 costimulation domains (data not shown).

We then tested whether additional cytokine support could improve CAR T cell activity. Indeed, when mice were implanted with U87 tumors overexpressing IL-2, EGFR806-CAR T cells completely eradicated all tumors without recurrence in the 90-day experimental window. All animals survived until 90 days, which is significantly longer than in EGFR806-CAR treated mice bearing non-IL-2 tumors (*P* =0.0359, log rank test) (*N* = 8 for each group, pooled data from 2 separate experiments).

### EGFR806-CAR T cells demonstrate a selective activation profile in a human iPSC teratoma model

**Figure 6:**
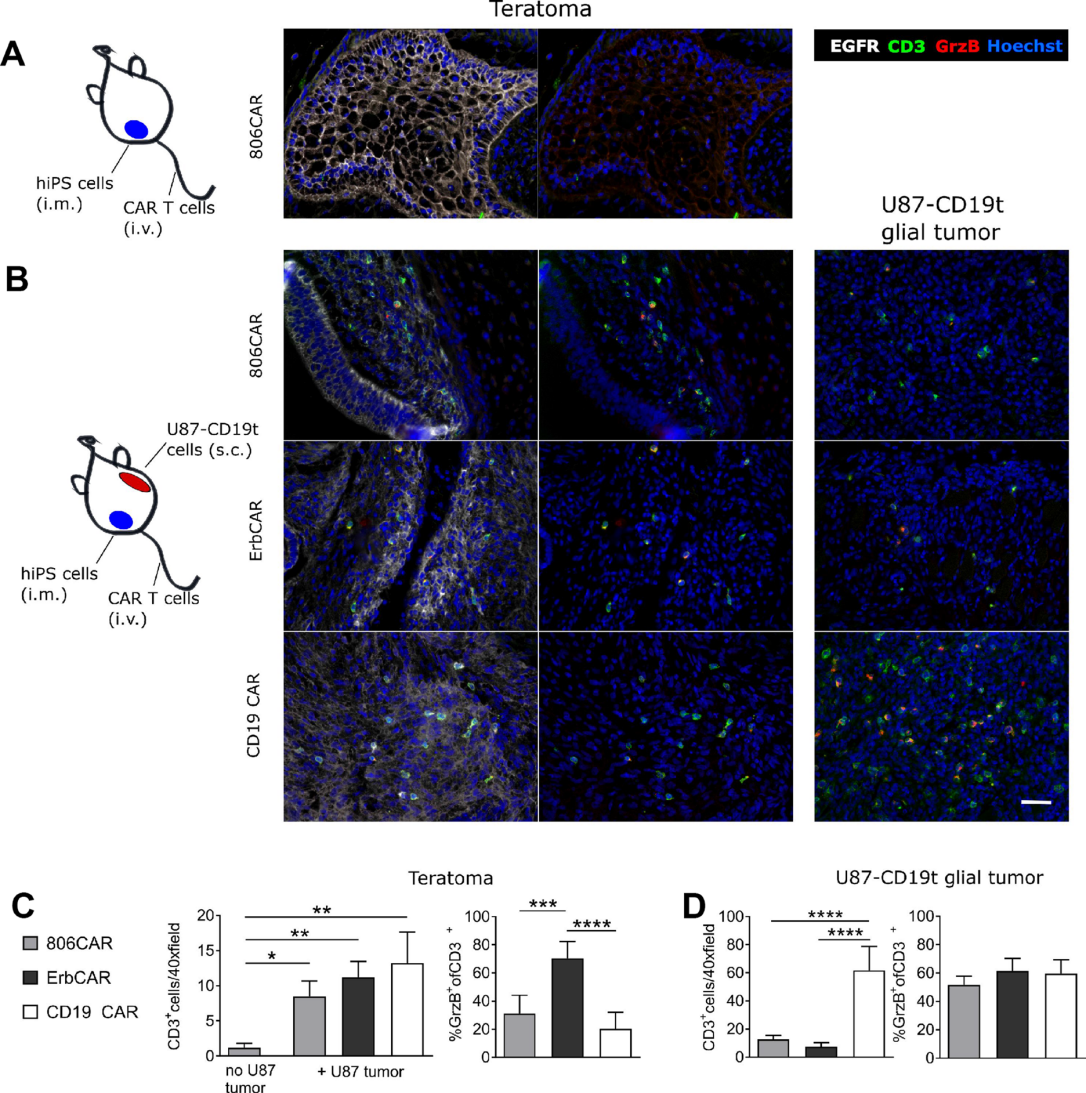
EGFR806-CAR T cells are selectively activated by tumor-specific EGFR expression in a human iPS cell teratoma model. (**A**) Teratoma-only model: mice were injected subcutaneously with human iPS (hiPS) cells in the left leg muscle, and EGFR806-CAR T cells were injected after teratoma has established. Immunolabeling of the teratoma (right 2 panels) shows minimal infiltration of CD3^+^ cells (green) into EGFR^+^ (white) regions of teratoma. (**B**) Teratoma-glioma model: mice were injected with hiPS cells in the left leg. After establishment of a palpable teratoma, U87 glioma cells expressing CD19t (U87-CD19t) were injected in the right flank. CAR T cells were infused 10 days after glioma cell injection. Representative images show immunolabeling demonstrating similar degrees of infiltration of CD3^+^ CAR T cells (green) into EGFR^+^ teratoma (left 2 columns) and EGFR^+^ U87-CD19t glioma (right column) after treatment with EGFR806 CAR (top), Erbitux CAR (middle) and CD19 CAR (bottom) T cells, with differing degrees of activation as indicated by Granzyme B (GrzB) labeling in red. (**C**) Left: infiltration of CAR T cells into teratoma is much lower in animals that did not receive concurrent U87-CD19t glioma grafts. Right: GrzB^+^ CAR T cells in EGFR^+^ teratoma as a percentage of total CD3^+^ teratoma resident T cells. Bars represent average cell number in 20 40× images from different EGFR^+^ tumor regions. Error bars, SEM. (**D**) Left: infiltration of CAR T cells into U87-CD19t glioma grafts on the other side of teratoma-bearing mice. Right: bars represent percentage GrzB^+^ CAR T cells of total CD3^+^ T cells in EGFR^+^ U87-CD19t glioma. Bars represent average cell number in 20 40× images from different glial tumor regions. Error bars, SEM. ^*^ *P <* 0.05; ^**^ *P <* 0.01; ^***^ *P <* 0.001; ^****^ *P <* 0.0001. Scale bar, 20 μm. All images were acquired at 40× magnification.

To evaluate the potential cross-reactivity of the EGFR806-CAR to wild-type EGFR in a live tissue assay, we developed an *in vivo* human iPSC xenograft teratoma CAR T cell activation model. In this model, we also assessed whether T cell activation by concurrent glioma changes the likelihood of on-target off-tumor toxicity. NSG mice were injected intramuscularly (i. m.) with human iPSCs and maintained untreated until palpable teratomas were established (45–60 days) (Figure 6A). One cohort of mice was then subcutaneously injected in the other flank of the animal with U87 EGFR-positive tumor cells expressing truncated CD19, followed by i. v. CAR T cell administration 10 days later (Figure 6B). Mice were treated with 2nd generation short spacer EGFR806-CAR, Erbitux-CAR (positive control which is expected to be activated by both glioma and EGFR-expressing teratoma), and CD19-directed CAR T cells (CD19-CAR, negative control that is only activated by the CD19-overexpressing glioma, but not the teratoma). We used a defined ratio of 1:1 CD4: CD8 product for the EGFR806-CAR infusions to reflect the composition of CAR T products in clinical development. Established teratomas and glial tumors were harvested 3 days post CAR T cell injection and analyzed by immunohistochemistry for T cell infiltration and activation in EGFR-positive regions of teratoma. Histological analysis of established teratomas by H&E staining demonstrated that multiple tissue types from different germ layer origins were established (Supplementary Figure 3). Parallel immunofluorescence analysis confirmed that teratomas regionally expressed EGFR, but never CD19, while the glial tumors retained uniform EGFR and CD19 surface expression (Supplementary Figure 4).

EGFR806-CAR T cells showed negligible infiltration of the teratoma when no concurrent glioma was present (Figure 6A). In mice bearing both teratoma and glioma, the rate of teratoma infiltration increased, and was similar for EGFR806-CAR, Erbitux-CAR, and CD19-CAR T cells (Figure 6B, 6C). However, teratoma-resident Erbitux-CAR T cells significantly upregulated Granzyme B (GrzB) relative to EGFR806-CAR and CD19-CAR T cells (*p <* 0.0001; Figure 6B, 6C). In contrast, there was no statistically significant difference in T cell activation (%CD3^+^/GrzB^+^) between Erbitux-CAR, EGFR806-CAR, and CD19-CAR T cells that infiltrated the CD19^+^EGFR^+^ U87 glial tumor mass (Figure 6D). The number of glioma-infiltrating Erbitux-CAR and EGFR806-CAR T cells was similar, but lower than that of the CD19-CAR T cells (Figure 6D). This difference may be due to higher CD19t expression levels compared to the intrinsic EGFR expression in the glioma cells, and is less likely due to differential expansion of the different CAR T cells, given their similar density in the teratomas.

These results lend additional support to the conclusion that EGFR806-CAR T cells have lower on-target, off-tumor activity than nonselective Erbitux-CAR T cells, as indicated by their minimal activation in wild type EGFR-expressing teratoma compared to Erbitux-CAR T cells. The on-target, on-tumor response is comparable between both CAR constructs, indicating that the difference in activation in the teratoma is not due to overall decreased activity of the EGFR806-CAR T cells.

## DISCUSSION

Designing an effective and safe CAR T cell therapy depends on choosing a tumor-specific target and tuning the signaling characteristics of the CAR. As the interaction of the extracellular domain with the target epitope is unique for each pairing, requirements for the extracellular spacer and intracellular signaling domains cannot be generalized across different CAR designs. We have developed a CAR based on the tumor-specific mAb806, which is selectively activated by tumor-expressed EGFR, be it full-length or the truncated EGFRvIII variant. Spacer length affected the efficacy of the EGFR806-CAR, with the short-spacer version showing the most robust *in vivo* tumor control.

We report consistent regression of orthotopic glioma in xenograft mouse models treated with EGFR806-CAR T cells, with an overall response comparable to other EGFR-directed CAR T cells (Erbitux-CAR, EGFRvIII-CAR, Nimotuzumab-CAR) on glioma xenografts [38, 40, 41]. We show that intracranial delivery is effective at controlling glioma growth in the brain, but the question of the optimal route of delivery is not yet settled in the field. In clinical trials of CD19-directed systemically delivered CAR T cells, small numbers of CAR T cells were detected in the cerebrospinal fluid [42, 43], however, it is unknown how efficiently they can traffic into the brain parenchyma. In clinical trials for glioblastoma, transient tumor regression has been achieved via intraventricular delivery [26], whereas systemic delivery, while safe, has not resulted in significant tumor response [15]. Certainly, many other factors, such as choice of epitope, CAR design, frequency of dosing, CAR T cell persistence, antigen expression, and antigen escape by the tumor are likely to be important for outcomes as well.

Affinity tuning of the CAR has been used as a strategy to reduce on-target off-tumor toxicity by using lower affinity CARs that spare normal tissue expressing low levels of the target [44–46]. For solid tumors, however, differences in CAR target expression levels throughout the tissue are the norm [47], which may decrease the efficacy of a low-affinity CAR. We thus did not rely on affinity tuning, and instead took advantage of the unique conformational properties of EGFR to ensure tumor specificity, as EGFR806-CAR binding is restricted to tumor-associated conformational states of the extracellular domains of EGFR. We ensured that EGFR806-CAR T cells are able to lyse tumor cells with a wide range of EGFR expression levels. We found that cytokine release and target cell killing by the EGFR806-CAR were similarly effective in all glioblastoma cell lines tested, and that it was active against EGFRvIII-transduced fetal human astrocytes, which expressed approximately 3000-4000 EGFR copies per cell. Other high affinity CARs require a minimum of 200-5000 cell-surface targets [48]. Glioblastoma expression of EGFRvIII is reported as several orders of magnitude higher [49], but decreased expression was reported upon treatment with EGFRvIII-directed CAR T cells [15]. Furthermore, we found that lower-level EGFR expression is common in glioblastoma. Therefore, we chose U87, the glioma cell line with the lowest EGFR expression level, for *in vivo* testing. Despite the EGFR806-CAR T cells’ robust *in vivo* activity, they did not show on-target off-tumor activation in EGFR-expressing teratomas, again confirming tumor specificity.

When used for CAR T cell targeting, the mAb806 scFv moiety remained highly specific for tumor-expressed EGFR, with no evidence of activity against native EGFR. *In vitro*, the EGFR806-CAR was minimally stimulated by normal fetal human astrocytes, but was activated by overexpressed EGFRvIII similarly to the nonselective Erbitux-CAR. With our novel human teratoma xenograft model, we have developed an efficient system for testing CAR T cell binding specificity in a wide variety of tissue types. EGFR806-CAR T cells only infiltrated EGFR-positive teratomas if the they were activated by a glioma at a different site in the same animal. However, the infiltrating EGFR806-CAR T cells did not become activated within the teratoma, unlike the nonselective Erbitux-CAR T cells. These findings suggest that teratoma infiltration may not be triggered by presence of local antigen, but rather by remote activation of the T cells. This is an intriguing observation given that lack of effector T cell infiltration into solid tumors has been a major obstacle in cell-based immunotherapy [50].

Important questions remain that cannot be easily answered in a xenograft mouse model. For example, how do the intracranially delivered CAR T cells traffic inside the CNS and what is the likelihood of escape into the systemic circulation? Human T cells are likely to behave very differently in the autologous host from which they were derived, and interaction with other tumor infiltrating immune cells will likely affect their performance. It is unknown whether CAR T cells can control infiltrative gliomas without injuring the surrounding brain parenchyma, and whether ablation of EGFR-expressing glioma cells will provide a selective survival advantage for EGFR-negative malignant cells.

Taken together, our data support the utility of targeting wild-type cell surface proteins on tumor cells that house a unique conformational epitope for selective CAR T cell targeting. The efficient and specific binding of the EGFR806-CAR supports its further development for clinical application.

## MATERIALS AND METHODS

### Generation of CAR constructs and lentivirus

The chimeric monoclonal antibody (mAb) 806 has been previously described [18]. Variable short (IgG4-hinge), medium (IgG4-hinge-CH3) and long (IgG4-hinge-CH2-CH3) spacer 2^nd^ generation 4-1BB-zeta CARs were constructed using the V_L_ and V_H_ segments of mAb 806 as previously described [44]. Each CAR sequence was linked to a T2A ribosomal skip sequence followed by a truncated EGFR (EGFRt) to facilitate selection of transduced cells. Second generation short spacer Erbitux-CAR T cells were produced by a similar mechanism, however the T2A sequence was followed by a truncated CD19 (CD19t). Lentiviral vector was produced in HEK 293T cells using the packaging vectors pCHGP-2, pCMV-Rev2, and pCMV-G [44].

### Generation of central memory T cell lines expressing EGFR806-CARs

CD8^+^CD45RO^+^CD62L^+^ central memory T cells (T_CM_) were isolated from peripheral blood mononuclear cells (PBMCs) of healthy donors (Puget Sound Blood Center, Seattle, WA) by negative selection using CD8 isolation kits and CD45RA microbeads and positive selection with CD62L microbeads (Miltenyi Biotec). Following isolation, CD8^+^ T_CM_ were stimulated with anti CD3/CD28 Dynabeads (Life Technologies) and transduced at a multiplicity of infection (MOI) of 3 on the third day of culture. EGFRt^+^ T_CM_ subsets were enriched by immunomagnetic selection using biotinylated Erbitux and anti-biotin microbeads (Miltenyi Biotec), then expanded as previously described [44]. CD8^+^ T_CM_ were maintained in RPMI medium supplemented with 10% fetal bovine serum, 2 mM L-glutamine, 50 IU/mL recombinant human interleukin 2 (IL-2) and 1 ng/mL recombinant human interleukin 15 (IL-15).

### Flow cytometry and immunophenotyping

Conjugated mAbs for CD3, CD4, CD8, CD45RO, and CD62L (Biolegend) were used for immunophenotyping. Tumor cell EGFR positivity and CAR construct expression, via the surrogate cell-surface marker EGFRt, was confirmed using biotinylated Erbitux (Cetuximab) and PE-conjugated streptavidin (SA-PE). CAR cell-surface and total expression was confirmed using biotinylated Protein-L (Genscript) and SA-PE or anti-CD247 (CD3z, BD Biosciences), respectively. Flow analysis was performed on an LSRFortessa (BD), sort-purifications on a FACSAriaII (BD) and data analyzed using FlowJo software (Treestar).

### Tissue microarray staining and analysis

Tissue microarrays containing duplicates of 33 glioblastoma cases, 2 brain anaplastic astrocytoma, and 5 normal brain tissue samples were ordered from US Biomax, Inc. (Cat: GL806c). Arrays were stained with primary mouse anti-human EGFR antibody (Clone 31G7, Invitrogen) at a 1:100 dilution. Briefly, slides were baked for 30 minutes at 60°C and deparaffinized on the Leica Bond Automated Immunostainer, followed by antigen retrieval with Proteinase K for 10min at 37°C. Blocking was performed for 10min at RT using Normal Goat Serum (10% in TBS) followed by primary antibody in Leica primary antibody diluent for 30min. Secondary antibodies (Life Technologies) were incubated with TMAs for 2hr at RT and diluted 1:500 in PBS with 0.2% BSA.

TMAs were imaged using the Nuance Multispectral imaging system (Perkin Elmer) on a Nikon Eclipse Ci upright microscope at 20 ×. Images were captured every 20 nm wavelength of light from 420 nm-720 nm. Data were analyzed by InForm analysis software (Perkin Elmer) using a threshold of 0.07OD.

### Cell line production and analysis

The A431, T98, U251T, U87 and Raji cell lines were obtained from the American Type Culture Collection (ATCC). Unless otherwise indicated, cell lines were maintained in DMEM or RPMI (Gibco) supplemented with 2 mM L-glutamine (Irvine Scientific; Santa Ana, CA), 25 mM HEPES (Irvine Scientific), and 10% heat-inactivated FCS (Hyclone). Total EGFR expression was analyzed by western blot analysis (Cat: sc-03, Santa Cruz Biotech) and antigen density was quantified by determining the anti-EGFR antibody (Clone EGFR.1, BD) binding capacity per cell using the BD QuantiBRITE system for fluorescence measurement [51]. Raji EGFR-vIII were generated by lentiviral transduction (EGFRvIII contains aa 1-29, 298-668 of EGFR) and enriched by immunomagnetic selection. U87 ffluc-IL2^+^ tumor cells were previously described [39].

### *In vitro* cellular assays

#### Chromium release assay

CAR T cell cytotoxicity was determined by chromium release assay. Target cells were labeled with ^51^Cr (Perkin Elmer), washed and incubated in triplicate with CAR T cells at various effector to target (E: T) ratios. Supernatants were harvested for g-counting 4 hours later and specific lysis was calculated using the standard formula [52]. Soluble EGFR peptide (aa 287-302) was purchased from GenScript for peptide inhibition assays.

#### Cytokine release assay

To investigate cytokine secretion, CAR T cells and target cells were plated at a 2:1 ratio and incubated for 24 hours. Supernatant was then analyzed for IL-2, IFNg and TNFa production using the Bio-Plex multiplex bead array system (Bio-Rad).

#### Orthotopic xenograft model and exogenous T cell transplantation

The orthotopic xenograft model was performed as previously described [39]. Briefly, 8-12-week-old adult male NSG mice were injected intracranially (i. c.) on day 0 with 2 × 10^5^ ffluc-IL2^+^ expressing U87 glioma cells 2 mm lateral, 0.5 mm anterior to the bregma and 2.5 mm deep from the dura. 7 days later, Mice were i. c. injected at the same site with a total of 2 × 10^6^ CAR T cells placed 2.5, 2.35 and 2.25 mm deep from the dura. Bioluminescent imaging was performed weekly by intraperitoneal (i. p.) injection of 4.29mg/mouse D-luciferin (Xenogen), anesthesia by isoflurane and imaging 15 minutes post D-luciferin injection using the IVIS Spectrum Imaging System (Perkin Elmer). Luciferase activity was analyzed using Living Image Software Version 4.3 (Perkin Elmer) and photon flux was analyzed within regions of interest. All animal experiments were approved by the Institutional Animal Care and Use Committee.

#### iPSC teratoma model and IHC analysis

To generate teratomas *in vivo*, 6-8 week old NSG female mice (Jackson Laboratories or bred in-house) were injected with 0.5 × 10^6^ human iPS cells prepared in a pro-survival cocktail [53] (generously provided by the Ellison Stem Cell Core of the institute for Stem Cell Regenerative Medicine at the University of Washington) via intramuscular (i. m.) injection into the inner left hind leg. Once teratomas became palpable (6-7 weeks), a subset of mice was subsequently engrafted with 5 × 10^6^ CD19t-expressing U87 glioblastoma tumor cells in USP grade PBS (Ameresco) and 50% matrigel (Corning) via s. c. injection into the right flank. Ten days post glioma inoculation, all animals received a tail vein injection of either USP grade PBS (Ameresco) vehicle alone or 50 × 10^6^ CAR T cells at a 1:1 ratio of CD4: CD8. All animals were euthanized 3 days after CAR T cell administration, perfused with PBS (Gibco) followed by 10% neutral buffered formalin (Thermo Scientific), and teratomas and subcutaneously engrafted tumors were collected for immunohistochemistry. IHC analysis was performed in EGFR^+^ regions.

#### EGFR teratoma immunohistochemistry

Slides were deparaffinized through a graded alcohol series, and antigen retrieval was performed using Digest-all 3 enzyme cocktail (Fisher, #3009) for 10 minutes at 37°C. Slides were blocked for 1 hour using 0.2% bovine serum albumin + 2% normal goat serum, incubated with primary antibody (clone 31G7, Fisher, #280005), diluted 1:100 for 1 hour in blocking buffer for 1 hour, RT. Slides were washed in PBS, and incubated in secondary antibody (Fisher #A-21240) diluted 1:500 in blocking buffer for 1 hour, RT. Slides were washed in ddH2O, and incubated with Hoechst dye (Fisher, #H3569) diluted to 2 μg/ml in ddH2O for 10 minutes. Slides were imaged on a Nuance Multispectral Imaging system on a Nikon Eclipse Ci microscope.

#### CD3 and Granzyme B teratoma immunohistochemistry

Slides were deparaffinized through a graded alcohol series, and antigen retrieval was performed using a decloaking chamber (Biocare medical, #DC2002) at 125°C for 3 minutes using Diva buffer (Biocare medical, #DV2004G1). Slides were blocked for 1 hour using 0.2% bovine serum albumin + 2% normal goat serum, incubated with primary antibodies (CD3 clone CD3-12, bio-rad #MCA1477, Granzyme B, rabbit polyclonal, LSBio # 34084), diluted 1:500 each for 1 hour in blocking buffer for 1 hour, RT. Slides were washed in PBS, and incubated in secondary antibody (Fisher #A-21240) diluted 1:500 in blocking buffer for 1 hour, RT. Slides were washed in ddH2O, and incubated with Hoechst dye (Fisher, #H3569) diluted to 2 μg/ml in ddH2O for 10 minutes. Slides were imaged on a Nuance Multispectral Imaging system on a Nikon Eclipse Ci microscope.

### Statistical analyses

Statistical analyses were conducted using Prism Software (GraphPad). Data are presented as means ± SD or SEM as stated in the figure legends. Student’s *t* test was conducted as a two-sided unpaired test with a confidence interval of 95% and results with a *P* value less than 0.05 were considered significant. Statistical analyses of survival were conducted by log-rank testing and results with a *P* value less than 0.05 were considered significant.

## SUPPLEMENTARY MATERIALS



## Abbreviations

CAR: chimeric antigen receptor
CNS: central nervous system
EGFR: epidermal growth factor receptor
IFNg: interferon gamma
iPSC: induced pluripotent stem cell
TNFa: tumor necrosis factor alpha
