# Supplementary material for: EGFR806-CAR T cells selectively target a tumor-restricted EGFR epitope in glioblastoma
Source: Oncotarget. 2019 Dec 17;10(66):7080–95. doi: 10.18632/oncotarget.27389 (PMC6925027; doi:10.18632/oncotarget.27389)
Supplement: Supplementary file 1 [file oncotarget-10-7080-s001.pdf]

EGFR806-CAR t cells selectively target a tumor-restricted EGFR epitope in glioblastoma

SUPPLEMENTARY MATERIALS

RESULTS

Estimating existence of EGFRvIII transcripts in tumor cell lines

The 5 cell lines processed for RNA-Seq analysis are listed in Supplementary Table 1. Numbers for mapped reads to the reference as well as only the EGFR locus (or engineered EGFRvIII) are shown in Supplementary Table 1. The epidermal cell line A431 has the highest relative expression of EGFR, which correlates with its high expression levels as measured by Western blotting and surface analysis (Figure 2). A431, T98, U251 and U87 cell lines express wild type EGFR but not EGFRvIII. Raji cells transduced with EGFRvIII served as controls and only expressed EGFRvIII and not wild type EGFR transcripts. As follows from Table S1, our alignment strategy sufficiently distinguishes reads mapping to EGFR versus EGFRvIII.

According to GRCh38.p12 annotation, the EGFR gene is composed of 59 exons that generate 11 transcript forms. Supplementary Figure 4 shows a schema of the 5' region of the EGFR locus. Concurrent exons were consolidated to yield exonic (or coding) regions that are partitioned by intervening introns.

TapeStation trace analysis of the RNA-Seq library region gave an approximate range of 200–720 bp. This includes sequencing adapters that add up to 121 bases, so the actual template sizes are between 80–600 bp. As can be seen from Supplementary Figure 4, EGFRvIII has a defining feature, in that it is the only transcript with exonic region 12 adjacent to region 1 (per GRCh38.p12 annotation). Major transcripts 1–6 also incorporate both regions 1 and 12; however, the intervening coding regions add up to 666 bases for transcripts 1 and 3, which skip region 11, and 801 bases for the others (3).

Sequence analysis was accomplished by capturing all reads originating in region 1 (upstream anchor reads) then interrogating if the corresponding mate pair reads mapped to region 12 (or further downstream). Supplementary Figure 5 shows the distribution of mate pair mappings to exonic regions. As observed, the highest proportions of mate pairs map to exonic region 5, mirroring the transition from region 1 to 5 for major transcripts 1–6 (Supplementary Figure 4). The progressive decrease in mate pair frequency downstream of exonic region 5 (regions 6 and 7) follows the template size distribution for sequencing, as expected. Given our template sizes sequenced, with a maximum of approximately 600 bp, it would be very unlikely for any fragmented template sourced from transcripts 1–6 to contain both regions 1 and 12 (or regions downstream of 12). Thus, any template featuring the expected transition from region 1 to 12 in EGFRvIII that would support the existence of this variant, would have been detected sensitively by our approach. Importantly, there is no evidence of such transitions from region 1 to 12 (Supplementary Figure 4), and therefore no detectable levels of EGFRvIII variant in any of the cell lines analyzed, using this extremely sensitive sequencing-based analysis.

MATERIALS AND METHODS

RNA-Seq

For each cell line, 5×10^6 cells were washed in PBS, pelleted, snap frozen and stored at −80°C. Total RNA was isolated using the RNeasy Mini kit (Qiagen) according to the manufacturer's recommendations and quantified with NanoDrop 2000 (Thermo Fisher Scientific, Waltham, MA). Total RNA integrity was assessed on TapeStation 4200 using R6K Screen Tape (Agilent Technologies Inc., Santa Clara, CA). RIN values obtained were above 9, indicating good RNA preparations. Additional quantitation for RNA-Seq library preparation was performed using a Trinean DropSense96 spectrophotometer (Caliper Life Sciences, Hopkinton, MA).

RNA-Seq libraries were prepared from total RNA with TruSeq RNA Sample Prep v2 Kit (Illumina Inc., San Diego, CA, USA). Library size distributions were validated using the D1000 Screen Tape on TapeStation 4200 (Agilent Technologies, Santa Clara, CA, USA). Additional library QC, blending of pooled indexed libraries, and cluster optimization was performed using Invitrogen Qubit® 2.0 Fluorometer (Life Technologies-Invitrogen, Carlsbad, CA, USA). RNA-seq libraries were pooled and clustered onto a S2 flow cell for sequencing on Illumina NovaSeq 6000 targeting a 110 × 90 base paired-end read length. Library preparation and sequencing was performed at the Fred Hutch Genomics Core, Seattle, WA.

Alignment of RNA-Seq reads

Genome sequence and comprehensive gene annotation pertaining for EGFR was obtained from GENCODE, Human Genome Reference GRCh38.p12 (https://www.gencodegenes.org/human/). The engineered EGFRvIII transcript sequence was appended to the genome reference. Sequenced reads were then aligned to this composite reference with STAR version 2.5.3a using a 2-pass mapping strategy [1, 2]. Starting from aligned reads in bam files, subsequent analysis was executed in R leveraging the Bioconductor framework (http://bioconductor.org/). Analysis was constrained to reads overlapping the EGFR gene on chromosome 7.

REFERENCES

1. Dobin A, Davis CA, Schlesinger F, Drenkow J, Zaleski C, Jha S, Batut P, Chaisson M, Gingeras TR. STAR: ultrafast universal RNA-seq aligner. Bioinforma Oxf Engl. 2013; 29:15–21. https://doi.org/10.1093/bioinformatics/bts635. [PubMed]
2. Dobin A, Gingeras TR. Mapping RNA-seq Reads with STAR. Curr Protoc Bioinforma. 2015; 51:11.14.1–19. https://doi.org/10.1002/0471250953.bi1114s51. [PubMed]
3. Sugawa N, Ekstrand AJ, James CD, Collins VP. Identical splicing of aberrant epidermal growth factor receptor transcripts from amplified rearranged genes in human glioblastomas. Proc Natl Acad Sci U S A. 1990; 87:8602–6. https://doi.org/10.1073/pnas.87.21.8602. [PubMed]
4. Garrett TP, Burgess AW, Gan HK, Luwor RB, Cartwright G, Walker F, Orchard SG, Clayton AH, Nice EC, Rothacker J, Catimel B, Cavenee WK, Old LJ, et al. Antibodies specifically targeting a locally misfolded region of tumor associated EGFR. Proc Natl Acad Sci U S A. 2009; 106:5082–7. https://doi.org/10.1073/pnas.0811559106. [PubMed]

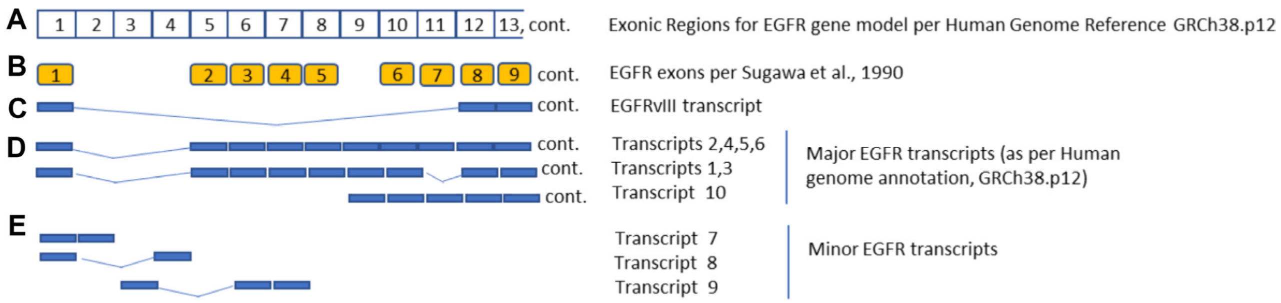

Supplementary Figure 1: Transcript map of the EGFR gene, 5' region. Line (**A**) shows the first 13 exonic regions from the most current genome build. In line (**B**), in order to orient to previous literature, exons corresponding to the "canonical" structure established by Sugawa *et al*. [3] are shown. The transcript map of the EGFRvIII variant, which features a deletion of the canonical exons 2 through 7 [3, 4] is depicted in line (**C**). The major and minor EGFR transcript forms annotated thus far are outlined in lines (**D** and **E**), respectively. Minor transcript 11 is not shown as it initiates further downstream, at exon 44. Downstream portions of EGFRvIII and major transcripts are also not shown (indicated as "cont." for continued).

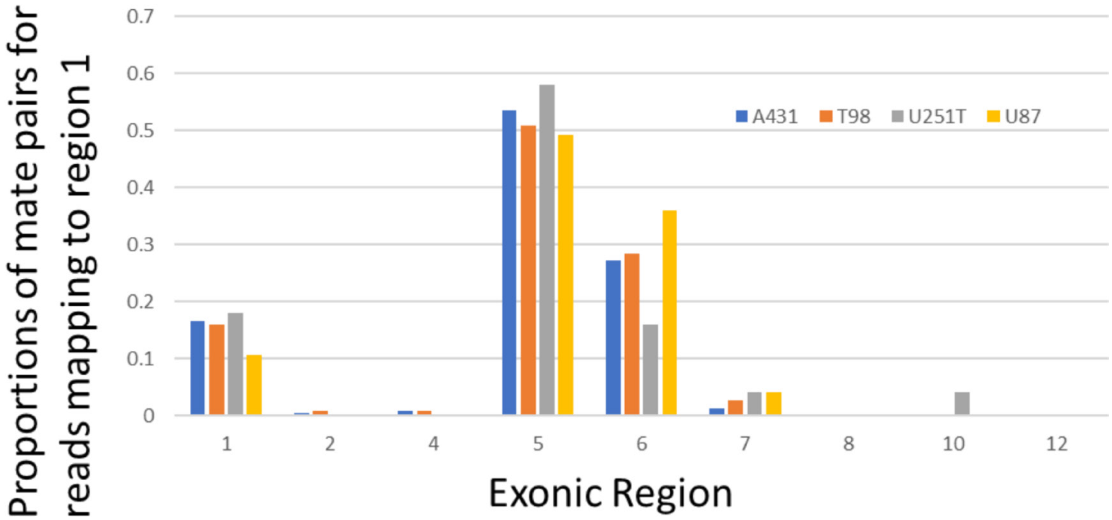

Supplementary Figure 2: Mate pair read mapping analysis of four glioma cell lines shows absence of EGFRvIII transcript. Bars show the distribution of anchor read mate pairs as proportions that map to different exonic regions.

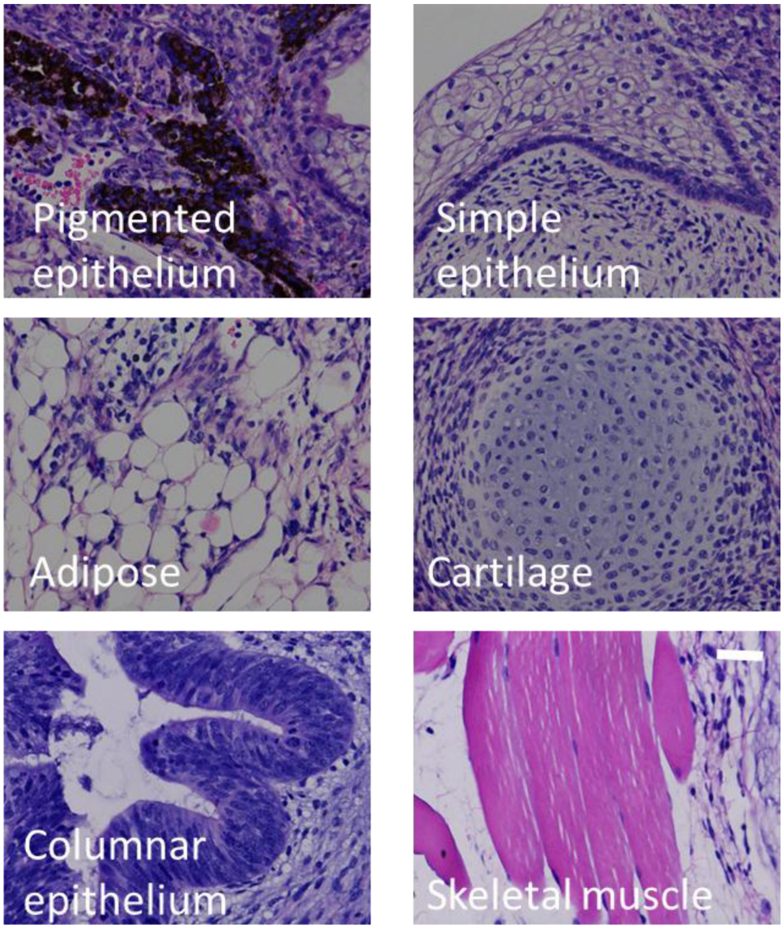

Supplementary Figure 3: Human iPSC-derived teratoma xenografts establish multiple tissue types from different germ layers. Composite structure H&E analysis of established teratoma. Scale bar 20 μM. Images were acquired at 40×.

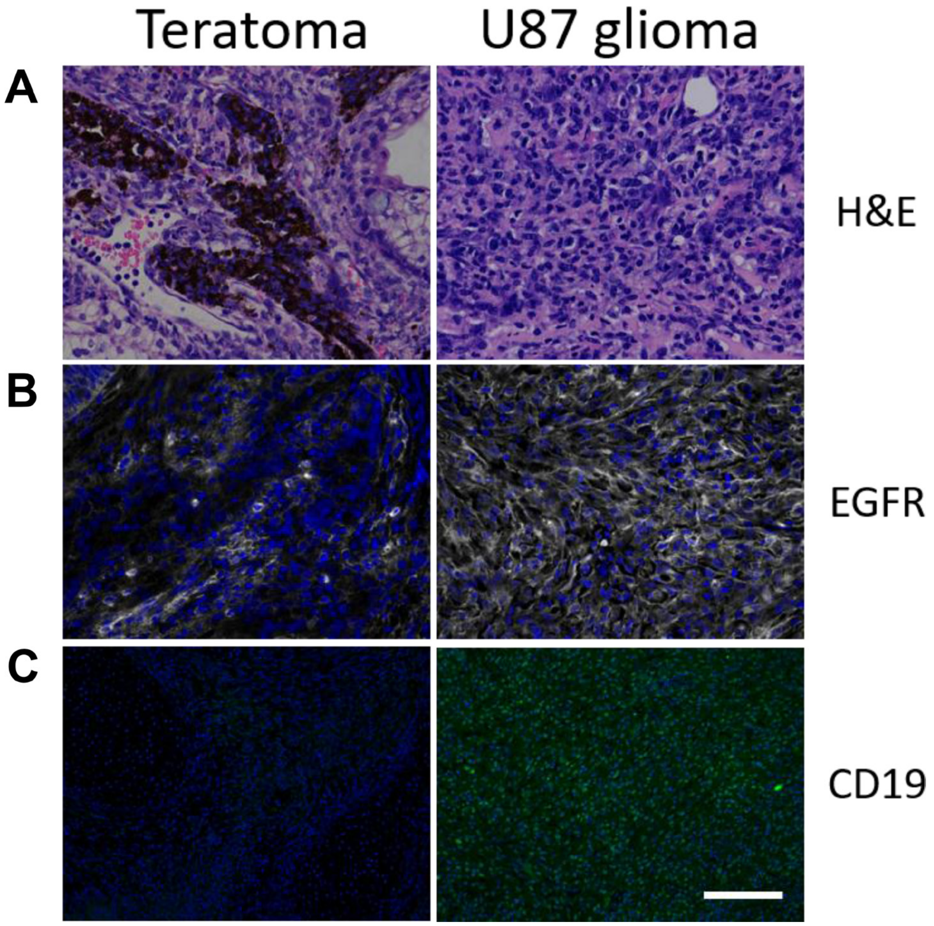

Supplementary Figure 4: Human iPSC-derived teratoma xenografts express EGFR but not CD19. Representative H&E analysis (top row) and immunofluorescence stains for EGFR (white, middle row) and CD19 (green, bottom row) of established teratoma and U87-CD19t glioma. Samples obtained 3 days post CAR T cell injection. Scale bar 40 μM. Images were acquired at 20×.
